# O14 Transitions in diagnosis and between services in juvenile idiopathic arthritis: living and learning

**DOI:** 10.1093/rap/rkaa054.002

**Published:** 2020-11-03

**Authors:** Rebecca Nock, Mohammed Akil, Lisa Dunkley, Rachel Tattersall

**Affiliations:** r1 The Medical School: The University of Sheffield, Sheffield, United Kingdom; r2 Rheumatology: Sheffield Teaching Hospitals NHSFT, Sheffield, United Kingdom

## Abstract

**Case report - Introduction:**

Juvenile Idiopathic Arthritis (JIA) is a lifelong condition in over 50% but there is limited knowledge of JIA in adulthood. We discuss C, who presented in childhood with polyarticular JIA which evolved into systemic lupus erythematosus (SLE). After careful transition she defaulted on care and had a life-threatening SLE flare. In adult services axial symptoms became dominant. Adult rheumatologists queried a third diagnosis of axial spondyloarthropathy, not recognising axial disease as a feature of JIA. This case highlights that knowledge sharing is needed to improve care of adults with JIA and reminds us that transfer can be a risky time.

**Case report - Case description:**

C presented age 11 with pain and swelling in the small joints of her hands and feet. She was treated for polyarticular JIA with methotrexate and etanercept.

At the point of transfer and having had early, detailed transition planning, C developed mouth ulcers, and Raynaud’s with lymphopenia, positive ANA, DsDNA, Ro, Sm, and RNP antibodies, low C4 and an elevated ESR, resulting in a diagnosis of evolving SLE/JIA. Treatment with methotrexate and etanercept continued but C’s adherence became poor. She stopped taking methotrexate due to nausea and ceased etanercept some months previously. C had extra transition support to enhance adherence and transferred into the young adult clinic (YAC) re-established on treatment.

After transfer, her SLE became more complex so there was further transition into the adult connective tissue disease (CTD) service. Azathioprine was prescribed but failed to control symptoms and was stopped, although adherence was a significant issue again.

C was admitted to hospital with an SLE flare causing rash, inflammatory arthritis, and nephritis with strongly positive DsDNA and low C3 and C4. She also had hyperferritinaemia, anaemia, leucopoenia, neutropenia, a raised CRP, and haemophagocytosis on bone marrow trephine due to haemophagocytic lymphohistiocytosis (HLH). This flare was a life-threatening culmination of poor adherence. C commenced mycophenolate and rituximab during this admission.

Engagement improved significantly but C developed back pain associated with a raised CRP. An MRI scan revealed evidence of bilateral sacroiliitis, interpreted by the adult SLE team as possible axial spondyloarthropathy, as she has a history of psoriasis. Referral back to the adolescent team highlighted that axial disease is a feature of polyarticular JIA. Close liaison between the two led to a revision of her diagnosis away from SLE to polyarticular JIA/SLE crossover syndrome with axial disease. C is currently managed with tocilizumab and mycophenolate.

**Case report - Discussion:**

Paediatric and adolescent rheumatologists recognise that inflammatory disease in young people may evolve: C went from a diagnosis of polyarticular JIA to SLE to possible axial spondyloarthropathy to polyarticular JIA/SLE crossover.

C transitioned into the YAC and then into the adult CTD service. There may have been a conflict here between focusing on generic young adult care, as opposed to specialist disease-related care. Defaulting on medical care is a normal phenomenon in adolescent development. For C this resulted in a life-threatening SLE flare. In adolescents, it is crucial to recognise transfer as a high-risk time, to remain alert to potential non-adherence and to chase up non-attendance especially immediately after seemingly successful transfer. Therefore, extra appointments and support may be needed in adult services.

JIA is a small yet significant part of general adult rheumatology workload. In Sheffield, JIA comprises 4% of the adult inflammatory arthritis service (compared with 39% for rheumatoid arthritis (RA), the commonest adult inflammatory arthritis). There is little in the literature about the phenotype or treatment of JIA in adults leading to the relative invisibility of JIA in adult rheumatology and relabelling of JIA as RA. Management of adolescent and adult JIA is further complicated by the differing definition and distributions of active joints between JIA and adult inflammatory arthritis. Both recognise tender and swollen joints as active, but in paediatrics, restricted joints are also ‘active’. Paediatric joint counts include all joints, not just selected joints as in, for example, DAS28 in RA. The burden of adolescent and adult JIA may be under-recognised and under-treated. For C this led to delayed recognition of axial disease in adult services, but early and close working of adolescent and adult specialists led to the correct diagnosis and reinforces the importance of taking a life course approach to JIA.

**Case report - Key learning points**
 

- C was managed in a seamless service where child-to-adult pathways are well developed. Despite this, she had significant non-adherence to medication, causing a life-threatening disease flare. The period immediately after transfer may be one of particular vulnerability and should be a target for extra support in adult services.
- Adult rheumatology is often super-specialised and just as transition from child to adult services may risk information being lost and a failure of developmentally appropriate care, transition within adult services (here from the YAC to the specialist CTD clinic) may also be challenging for patients and professionals alike. What worked well here was referral back to the YAC when axial disease was recognised and the shared approach to diagnosis and appropriate management.
- The optimum way to assess JIA activity in adults is not established. There is evidence that using scores for rheumatoid arthritis such as DAS28 risks underestimating JIA activity and denying young people access to treatment but there are no validated scores for adult JIA. This is a key area for future research.
- JIA persists into adult life in at least half of those with childhood onset disease.
- It is important that all rheumatologists appreciate the course, impact, and phenotypes of JIA in adults, to ensure the best outcomes and treatment. Young adult clinics are an ideal way to ensure developmentally appropriate care, but may not be feasible in all services, making education about JIA for adult rheumatologists (which paediatric and adolescent rheumatologists are ideally placed to deliver) all the more necessary.
- Young people with JIA need to be continually educated about their disease especially at transfer to adult services so they can remind adult rheumatologists that they have JIA.
- Good collaboration across all areas of rheumatology improves care for children, adolescents, and adults with rheumatic disease

